# Supplementary material for: The Effect of Dietary Supplementation of Crocetin for Myopia Control in Children: A Randomized Clinical Trial
Source: J Clin Med. 2019 Aug 7;8(8):1179. doi: 10.3390/jcm8081179 (PMC6724222; doi:10.3390/jcm8081179)
Supplement: Supplementary file 1 [file jcm-08-01179-s001.pdf]

Supplementary Information for

The Effect of Dietary Supplementation of Crocetin for Myopia Control in Children: A Randomized Clinical Trial

Kiwako Mori[1,2,4], Hidemasa Torii[1,2,4], Satoko Fujimoto[3,4], Xiaoyan Jiang[1,2], Shin-ichi Ikeda[1,2], Erisa Yotsukura[1,2], Shizuka Koh[3], Toshihide Kurihara[1,2,*],Kohji Nishida[3,*], Kazuo Tsubota[1,*]

Supplementary Table 1

**Table S1**	**Safety**					
Subject	69 patients（male：38 patients, female：31 patients）					
Adverse reactions	0 patient 0 case					
Adverse events	30 patients 50 cases（All: Not Related）					
	Influenza A infection	2	Cold	9	Fracture（Left ankle）	1
	Influenza B infection	8	Itchy eyes	1	Parotitis	1
	EB virus infection	1	Bronchitis	1	Headache	1
	Allergic conjunctivitis	2	Seasonal allergic rhinitis	1	Fever	2
	Virus conjunctivitis（left eye）	1	Acute conjunctivitis	1	Sprain（right big toe）	1
	Osteochondrosis of tarsus	1	Acute conjunctivitis（left eye）	1	Streptococcal infection	2
	Osteochondrosis of tarsus（left heel）	1	Acute upper respiratory infection	1	Nausea	1
	Gastroenteritis	1	Acute sinusitis	1	Cervical lymphadenopathy	1
	Sore throat	2	Acute chalazion（left eye）	1	hordeolum（right eye）	1
	Infectious gastroenteritis	1	Stomatitis	2		
Withdrawn	2 patients. Less intake (n = 1), inconvenient (n = 1)					
